# Acceptability and use determinants of digital health technologies for HIV services: a qualitative study of emergency care patients in Nairobi, Kenya

**DOI:** 10.3389/fdgth.2025.1697814

**Published:** 2026-01-23

**Authors:** Joshua Smith-Sreen, Benson Timothy, Beatrice Ngila, John Wamutitu Maina, Sankei Pirirei, John Kinuthia, David Bukusi, Harriet Waweru, Rose Bosire, Carey Farquhar, Michael J. Mello, Adam R. Aluisio

**Affiliations:** 1 Warren Alpert Medical School of Brown University, Providence, RI, United States; 2 Kenyatta National Hospital, Nairobi, Kenya; 3 Department of Research & Programs, Kenyatta National Hospital, Nairobi, Kenya; 4 Department of Global Health, University of Washington, Seattle, WA, United States; 5 Center for Public Health Research, Kenya Medical Research Institute, Nairobi, Kenya; 6 Department of Emergency Medicine, Alpert Medical School of Brown University, Providence, RI, United States

**Keywords:** Africa, digital health, emergency medicine, HIV testing services, Kenya

## Abstract

Digital health technologies (DHTs) represent a promising strategy to improve access to HTS (HIV testing services), particularly among underserved higher-risk populations often missed by current programming, including young adults under 25 years. In 2017, Kenya's Ministry of Health introduced BeSure™, a DHT providing information on HIV, self-testing, and facility geo-location. Given increased risks for HIV among injured populations, this study assessed the acceptability of BeSure™ as a DHT for enhancing HTS in a Kenyan emergency department. Using purposive sampling, participants were provided a brief description of the tool BeSure™ and then completed in-depth interviews using a semistructured guide between August and November 2023. Deductive and inductive analyses were applied using a codebook based on a published framework for healthcare intervention acceptability, examining core themes of affect, burden, ethicality, coherence, opportunity cost, and perceived effectiveness. Among 24 participants, the median age was 25, half were female, and 58% had achieved secondary education or below. Few participants (21%) were aware of BeSure™ prior to data collection. Barriers to awareness included limited marketing of the tool and apathy toward health-related matters. However, strategic advertisement within healthcare encounters and through social media platforms including TikTok and Facebook (especially for young adult participants) could facilitate awareness. Barriers to potential use include low access to technology in rural communities, persisting stigma toward HIV, and low perceived HIV risk (especially among older participants). Despite these barriers, participants across age groups found the tool widely acceptable across the predetermined domains. These qualitative data highlight the acceptability of DHTs for HTS enhancement among injured populations in Nairobi, Kenya. Findings underscore the limited awareness of BeSure™ among this higher-risk population, suggesting that targeted advertisement, demand creation, and stigma reduction strategies are critical to successful implementation of these technologies.

## Introduction

HIV and physical injury impose significant health burdens in low- and middle-income countries (LMICs), with particularly high incidences reported across African nations (1). Those most likely to need emergency care for injuries in general are also persons at risk for HIV acquisition, including young adults and males, commercial sex workers, men who have sex with men, people who inject drugs, and transgender persons, collectively referred to as key populations at risk for HIV (2–6). Studies have demonstrated that injured populations have higher HIV prevalence than the general population in African countries such as Kenya (7, 8), highlighting the potential utility of emergency care settings for HIV testing services (HTS). A prospective study completed in 2021 at the Kenyatta National Hospital (KNH) found that fewer than 6% of injured persons treated in the emergency department (ED) were offered HTS; however, among those tested, approximately10% were newly diagnosed persons living with HIV (PLHIV) (9, 10). Although universal HTS screening is recommended during health facility encounters in Kenya, implementation in EDs has not yet been achieved (11, 12).

Digital health technologies (DHTs) designed to improve access to HTS, particularly HIV self-testing, have been successfully implemented globally (13). In Kenya, DHTs may be particularly useful in contemporary HIV prevention programs, as the epidemic is increasingly concentrated among higher-risk populations who face more barriers to accessing health services and require innovative targeting strategies (14). Such higher-risk populations are more likely to present to EDs, making these venues appropriate settings for targeted HIV programming (15). In Kenya, the Ministry of Health has identified priority populations for HIV testing and prevention efforts, including young adults aged 18–24 years (16). Despite this, HIV prevention programming in the region has neglected this age group (17), which is estimated to account for 38% new infections in Kenya. As such, digital technologies implemented in settings that provide care to persons with higher-risk profiles for HIV (15), like the ED, may have the potential to direct resources to priority populations such as young adults. However, no prior studies have examined such technologies, specifically within emergency care populations in Kenya. Indeed, Kenya’s digital health policy landscape supports this approach. The Digital Health Act of 2023 established a new agency tasked with integrating and governing e-health within routine service delivery (18).

One example of such technology is BeSure™, a mobile and web-based HIV-focused digital health technology, introduced in 2017 by Kenya's National AIDS and STI Control Programme (NASCOP), in collaboration with local and international partners. Anecdotally, its utilization is thought to be low (19). BeSure™ was maintained as an open-access resource until mid-2023, at which time it was taken offline for redesign and updating (19). The platform provided HIV testing and treatment information, map-based locations for HIV care sites, and guidance around test results throughout Kenya. The BeSure™ interface also offered multiple follow-up options—including toll-free calls, text messages, and WhatsApp™—to connect clients with trained clinical staff for support. Despite the potential of technologies like BeSure™ to streamline HIV prevention and testing, evidence on their utilization, integration into health services, and their impact on clinical outcomes remains limited (20). This study therefore aimed to explore the acceptability of the BeSure™ as an example DHT for enhancing HTS among emergency care patients in Nairobi and to identify determinants of DHT use for HTS in this care setting.

## Methods

### Design and sampling

**Figure 1:**
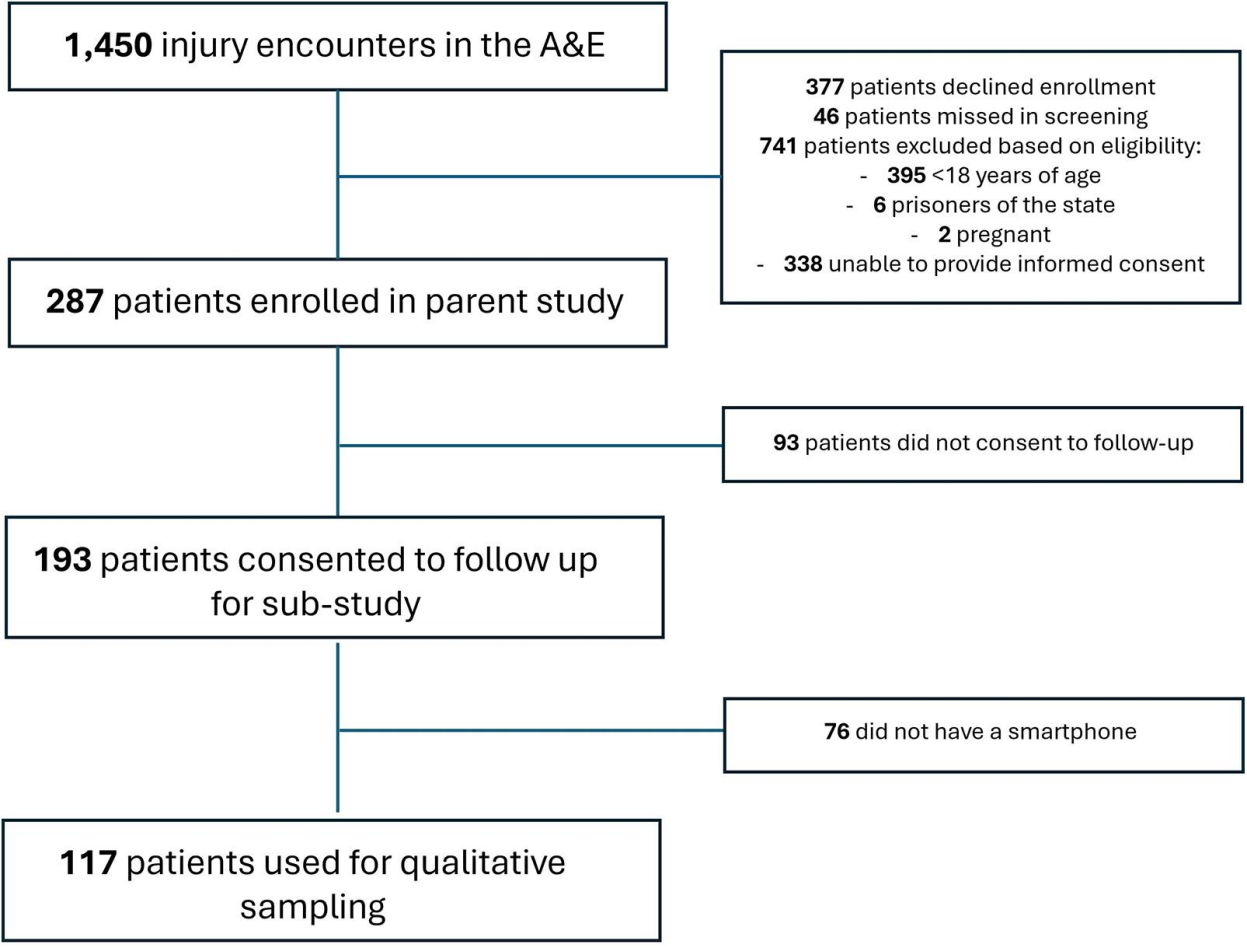
Eligibility diagram describing substudy sampling from parent study population, August–November 2023.

The target population included adults presenting to the KNH emergency department for physical injury, enrolled in the parent study assessing implementation of the HIV Enhanced Access Testing in Emergency Department (HEATED) program in Nairobi, Kenya, which commenced prior subsampling (21). This program was an institutional and behavioral intervention to improve HIV testing among injured patients in the emergency department. Among patients who consented to follow-up contact, purposive sampling was applied to yield equal distribution across gender and age strata (younger or older than 25 years). These strata were selected to capture potential differences in perceptions across gender and to reflect the prioritization of young adults as a key population for HIV services in Kenya (16). Inclusion criteria from the parent study were applied: Participants had to be older than 18 years old, non-pregnant, not incarcerated, and had consented to further contact in the parent study. In addition, only participants reporting access to a smartphone were included in interviews to enable meaningful discussion of digital health technologies, including smartphone-based applications. Participant sampling from the parent study population, using eligibility criteria, is further described in Figure 1. Selection bias introduced by this criterion is acknowledged; however, findings are expected to remain generalizable given high smartphone penetration across Kenya and the fact that smartphone users represent the application's target audience.

### Data collection

Eligible participants were randomly selected from the subsample, screened for consent, and enrolled via telephone. Only patients who had provided consent to follow-up contact in the parent study were approached for this qualitative study. Consenting participants were scheduled for in-depth interviews (IDIs) at a convenient and confidential location at the hospital. Participants received a compensation of 1,000KSH.

Prior to each IDI, written consent was obtained, the research question was explained, and participants were provided a pamphlet of the BeSure™ application in English and Kiswahili (Supplementary Material 1). As the BeSure™ tool was offline in 2023, the actual application was unavailable; participants were interviewed on its concept after description on its features. A semistructured guide was developed based on Sekhon and colleagues' prospective theoretical acceptability framework (TFA), which draws on psychology-grounded constructs developed systematically based on scoping review of studies evaluating healthcare intervention acceptability, effectiveness, and tolerability (22, 23). The guide included sections on barriers and facilitators to awareness of digital health technologies, determinants of BeSure™ use, and perceptions of DHTs more broadly to contextualize responses and mitigate threats to generalizability posed by the tool’s offline status (Supplementary Material 2).

IDIs were conducted between August and December 2023 by a Kenyan research team member with training in qualitative methods and fluent in both English and Kiswahili, with a notetaker present. Interview debriefs were reviewed by the study team within 1 day to determine additions to the codebook or challenges in data collection. Interviews were conducted until thematic saturation was achieved, as determined by the interviewer, notetaker, and data analysis team after reviewing debriefs based on constant comparison to existing codes, until at least three debriefs had no new themes identified. A unique identification number was used for all qualitative data collection and analysis activities. Interview recordings were translated into English and transcribed manually. Transcripts were imported into Dedoose Version 9 software for management and coding.

### Coding and analysis

A codebook was developed using *a priori* themes for deductive analysis. These themes were generated based on hypothesized barriers and facilitators to tool use, as well as Sekhon and colleagues' prospective theoretical acceptability framework (TFA)—a psychology-grounded construct systematically developed through a scoping review of studies evaluating healthcare intervention acceptability, effectiveness, and tolerability (22, 23). Themes incorporated into the codebook included affect, burden, ethicality, intervention coherence, opportunity cost, and perceived effectiveness. Each theme represented a parent node to which codes were attributed in the analysis, and each parent node had an operational definition to be used by coders. Affect was defined as positive or negative feelings expressed about the digital health tool. Burden was defined as any description of high or low perceived effort in using the digital health tool. Ethicality was defined as alignment of the tool with patient values. Intervention coherence was defined as recognition of the digital health tool's intended outcomes to improve access to HTS. Opportunity cost was defined as losses attributed to use of the digital health tool. Finally, perceived effectiveness was defined as the digital health tool being expected to achieve improved access to HTS.

Inductive and deductive thematic analyses were conducted on IDI transcripts. *A priori* themes were extracted based on the original codebook and categorized according to the sampling strata. Themes emerging during the coding process were added to the codebook for inductive analysis. Study personnel reported themes that emerged while coding a transcript, which were then discussed between two study members until consensus was reached before inclusion in the codebook. One quarter of transcripts were coded independently by two trained research study personnel and concordance analysis was conducted for internal validity of the codebook. Once >85% concordance was achieved, one trained research study personnel completed coding for the subsequent transcripts.

### Ethics

The study was approved by the University of Nairobi Ethics and Research Committee (Protocol Number: P667/08/2022) and the Institutional Review Board of Rhode Island Hospital (Protocol number: 1953237-1).

## Results

### Participant demographic profiles

This qualitative study sampled 24 participants who had been treated in the KNH ED. The sample comprised 12 males and 12 female participants, aged between 18 and 42 years. More than two-thirds of the participants were aged between 18 and 29 (*n* = 17, 70.8%). All participants reported use of and familiarity with smartphones and mobile applications. Participants provided their insights and perceptions regarding digital health applications in HIV-related services, particularly the use of the BeSure™ application to enhance testing and other services in Kenya. Table 1 presents the demographic profiles of the participants.

**Table 1:** Demographic and clinical characteristics of interviewed population.

Demographic characteristic	Proportion of participants (*n*, %)		
	Overall *n* = 24	Older adult *n* = 12	Young adult *n* = 12
Age (median, IQR)	25 (23.0, 30.0)	30 (28.8, 33.8)	23 (20.8, 23.0)
Gender (*n*, %)			
Female	12 (50.0)	6 (50.0)	6 (50.0)
Male	12 (50.0)	6 (50.0)	6 (50.0)
Relationship status (*n*, %)			
Monogamous	2 (8.3)	1 (8.3)	1 (8.3)
Polygamous	1 (4.2)	1 (8.3)	0 (0.0)
Married	7 (29.2)	6 (50.0)	1 (8.3)
Separated (still married)	1 (4.2)	0 (0.0)	1 (8.3)
Single	12 (50.0)	3 (25.0)	9 (75.0)
Wishes not to disclose	1 (4.2)	1 (8.3)	0 (0.0)
Education level achieved (*n*, %)			
Beyond secondary school	10 (41.7)	5 (41.7)	5 (41.7)
Primary school	1 (4.2)	1 (8.3)	0 (0.0)
Secondary school	13 (54.2)	6 (50.0)	7 (58.3)
In school (*n*, %)			
No	21 (87.5)	12 (100.0)	9 (75.0)
Yes	3 (12.5)	0 (0.0)	3 (25.0)
Current employment (*n*, %)			
No job/not working currently	6 (25.0)	0 (0.0)	6 (50.0)
Self-employed	6 (25.0)	3 (25.0)	3 (25.0)
Work laborer	9 (37.5)	6 (50.0)	3 (25.0)
Working professional	3 (12.5)	3 (25.0)	0 (0.0)
Has a primary care provider (*n*, %)			
No	17 (70.8)	8 (66.7)	9 (75.0)
Yes	7 (29.2)	4 (33.3)	3 (25.0)
Never previously tested for HIV (*n*, %)			
No	4 (16.7)	0 (0.0)	4 (33.3)
Yes	20 (83.3)	12 (100.0)	8 (66.7)
Condomless sex in the last 6 months (*n*, %)			
No	13 (54.2)	6 (50.0)	7 (58.3)
Wishes not to disclose	2 (8.3)	0 (0.0)	2 (16.7)
Yes	9 (37.5)	6 (50.0)	3 (25.0)
Missing	1 (4.2)	1 (7.7)	0 (0.0)
Aware that HIV self-tests exist (*n*, %)			
No	5 (20.8)	2 (16.7)	3 (25.0)
Yes	19 (79.2)	10 (83.3)	9 (75.0)

Participants aged 24 years and older were more likely to be working (100%) compared to younger participants (50%). The majority of the participants had achieved a secondary school level of education (54%). In terms of clinical history, the majority of the participants did not have a primary care physician (67%), had previously been tested for HIV (83%), did not have any medical conditions (87.5%), and had not engaged in condomless sex in the last 6 months (54%). The majority of the participants were aware of the existence of HIV self-tests (79%).

### Facilitators and barriers to awareness

#### Systemic and structural

The majority of the participants reported being unaware of the BeSure™ DHT (80%), but identified both facilitators and barriers to awareness. Many participants noted that greater advertisement would have increased their awareness of the digital health tool. Preferred modes of advertisement included promotion at health facilities where patients received care [Code frequency (CF) = 42] and social media marketing of the tool (CF = 41). All young adult participants (100%) specifically highlighted the use of social media platforms, such as Instagram™ and TikTok™, as a useful mode (compared to 75% of older participants).

“You know in this generation, it works better if they could take the whole aspect to Instagram, TikTok and campaign about it; talk to students and the elderly and let them know about (the tool).” (Participant 23, Female 23 years old)

Suboptimal advertisement was highlighted frequently as a barrier to awareness (CF = 9). Multiple participants raised concerns around marketing to patients presenting for acute injury, given challenges engaging or remembering in this setting.

“It’s somehow hard to remember (the app) because at that time (in the hospital) I was in pain such that I was straining to answer the questions.” (Participant 9, Male 39 years old)

Others reported that healthcare settings may serve as effective venues for education and awareness campaigns (CF = 42), providing that targeting is strategic, regarding where in a facility (e.g., in the wards), when in the care process (e.g., after treatment and stabilization), who on the care team (e.g., providers actively involved in care or department heads), and the how the communication occurs (e.g., providers taking a gentle approach to patients).

“(Patients) will have to stay for some time, so you guys can talk to them during that time while they are in the wards.” (Participant 21, Male 25 years old)

“When someone is still at the A&E, first thing is they’re scared and terrified. So I think, it would be better to actually walk arid the wards and try create awareness around there. I believe most patients in the wards have recovered from their initial shock, stabilized a little and can listen or pay attention.” (Participant 2, Female 23 years old)

“You can reach them through the dispensaries in those areas so that before or after treatment, they are told about the app and what it can do.” (Participant 18, Male 23 years old)

“You can appoint people to create awareness in different times so that even when the patient wakes up even from coma, there will be someone to educate them.” (Participant 14, Male 25 years old)

“Yeah, in the hospital you can use the departments to talk to people, maybe by the head of departments, though it might get a little hard in the casualty department because maybe someone is in pain, but some will agree to talk. But I think in the hospital, the department can help by telling the members of staff. Or the nurses who are there nursing them, they are the right people, or the doctors. You can use them because as they administer treatments and tests, they can be able to talk to the patient, and they will be ready since they are in pain, they will just listen to the doctor.” (Participant 6, Female 36 years old)

Some participants indicated that structural or government-level failures created barriers in awareness to such applications, suggesting that the responsibility may extend beyond healthcare institutions to the systems level. On the other hand, one participant expressed skepticism that high-level government intervention would effectively address the lack of awareness of such tools, alluding to a lack of trust in the Kenyan government.

“Maybe it’s the health sector that is quiet about it; the blame is on the health sector.” (Participant 7, Female 29 years old)

“I think we don’t need any external input, because if the government gets involved people will think it’s a scam and run away from it…we don’t need the politicians to chip in.” (Participant 23, Female 23 years old)

#### Interpersonal

Interpersonal barriers to awareness include apathy toward health-related topics (CF = 9), which limited interest in or attention to digital technologies focused on HIV prevention. Older adults highlighted this apathy more frequently (33% compared to 17% in young adults).

“Others would see no importance in (the digital health tool), they’ll be like…so what?” (Participant 6, Male 36 years old)

#### Facilitators and barriers to use

None of the interviewed participants had previously used the BeSure™ DHT, but they identified potential barriers and facilitators to its use as an example digital health technology.

#### Systemic and structural

Access to technology was the most frequently reported theme mediating potential use of a digital health tool (CF = 19 as a facilitator, CF = 30 as a barrier). Owning a smartphone was not commonly reported as a barrier, as participants highlighted the widespread prevalent of smartphones in Kenya. Participants specifically highlighted how digital literacy was common among young populations, with high smartphone penetration helping to overcome systemic barriers to use. Rather than access to smartphones, participants reported access to data and the internet as barriers to using a digital health technology. In addition, while young populations were highlighted as digitally literate, rural populations were described as facing barriers in using digital health technologies.

“The youth bracket mostly and all of them know how to operate a phone.” (Participant 3, Male 24 years old)

“Most people are illiterate, and some can have hard time going through notice boards and things like that, but almost everyone nowadays owns a smartphone.” (Participant 14, Male 25 years old)

“For somebody who has a smartphone and somebody who has the space but doesn’t have the data might have a challenge” (Participant 4, Female 23 years old)

“Yes, people living in rural areas who are not used to operating smartphones (will face challenges).” (Participant 17, Female 23 years old)

#### Interpersonal

Interpersonal influences and beliefs were also reported as mediators of potential use of a digital health tool. Stigma concerns (CF = 16) were raised as interpersonal barriers to engaging with a phone-based tool focused on HIV, even after participants were primed with information BeSure™ as an example DHT (Supplementary Material 2). Apathy (CF = 14) was reported as another interpersonal barrier, reflecting limited interest in engaging with a health-related digital tool even when awareness was present.

“HIV is associated with a lot of stigma and it would be hard for people to engage in such issues publicly.” (Participant 20, Male 22 years old)

“No, I don’t think (internet access is an issue). Because people look for bundles from anywhere when they want to do other things. So, (internet access) is not an excuse; maybe some people don’t use because they don’t want to.” (Participant 9, Male 39 years old)

Compared to older participants, young participants reported high perceived HIV risk as a facilitator to tool use (CF = 2). Conversely, older participants more commonly reported low perceived HIV risk as a barrier (CF = 4).

“Yeah, it’s good because some of us have trust issues when it comes to our partners.” (Participant 25, Female 20 years old)

“Maybe most of the people are afraid of knowing their status. It’s like you get out there and tell someone you want to test them for HIV, they will back off.” (Participant 9, Male 39 years old)

#### Acceptability

**Figure 2:**
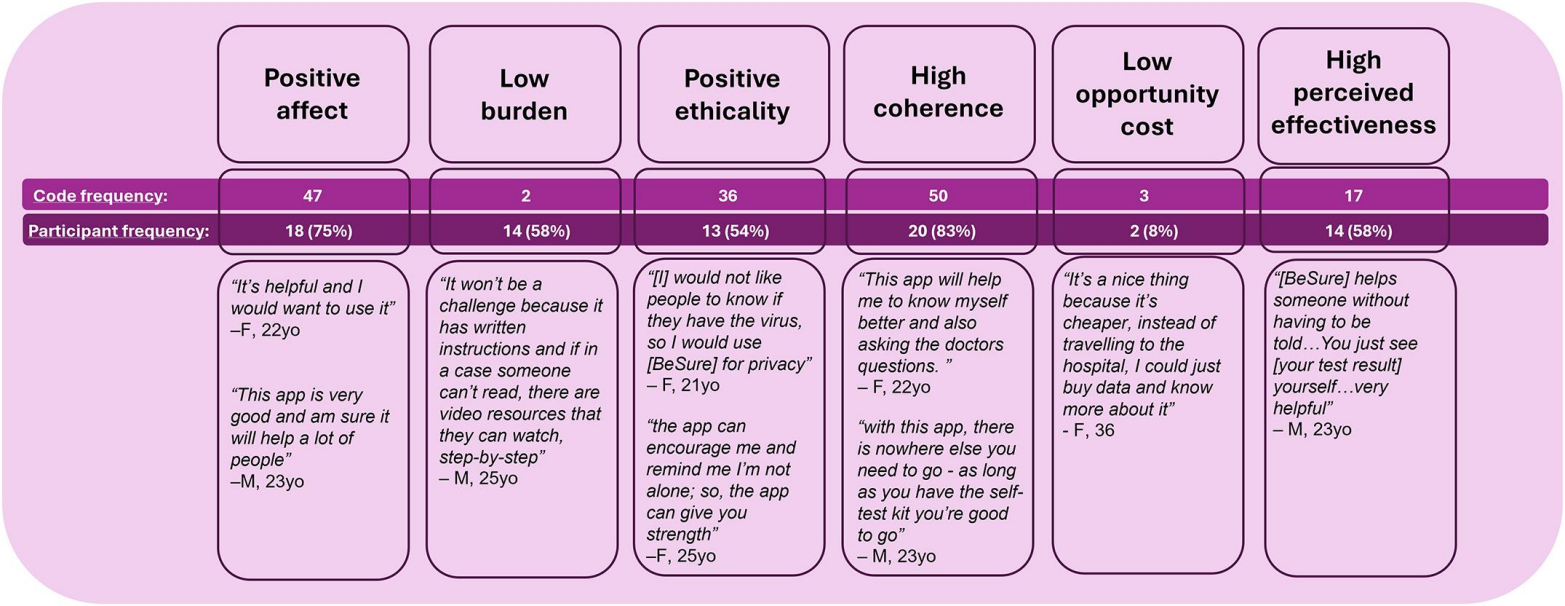
Thematic mapping acceptability of BeSure™ digital health technology for HIV service enhancement among emergency care patients at Kenyatta National Hosptial, August–November 2023.

Acceptability themes extracted and coded from participant interviews are displayed in Figure 2. After learning about BeSure™ as an example of a digital health technology for HIV service enhancement (Supplementary Material 2), the majority of the participants (CF = 47, 75% participants) made positive remarks about the tool, indicating their positive affect. More young adults made positive remarks (83%) about the tool than older participants (67%).

“To me personally…when I have this app it would be easier to identify pharmacies… I think it’s a good app because it does not disclose your identity, and you’ll only need to contact the professional advice you on what to do.” (Participant 5, Male 22 years old)

“(BeSure) is a good one, it can really help, sometimes you want to know about your status or maybe you have the virus, it can help, advice and encourage you and I love it.” (Participant 19, Female 31 years old)

The tool aligned well with participant ethicality or values (CF = 36, 54% participants), particularly around healthcare for HIV, including privacy, anonymity, convenience, and cost. Participants reported how this digital tool for HIV testing addresses persistent HIV stigma by preserving anonymity and privacy. Others alluded to how the digital tool could mitigate the fear of being diagnosed with HIV in a formal patient encounter or how it could help empower users by connecting them to care resources. Alignment with values was reported across younger and older participants.

“I would use of because of the privacy; since there are people who HIV is not a big deal to them and there are others who would not like people to know if they have the virus.” (Participant 22, Female 21 years old)

“Maybe after testing I find out that maybe the results are not good; so, maybe you can go back to the app again and talk to a professional. So, it comes in handy and help someone without having to be told or forced. You just see it yourself. For I see it as very helpful at that point.” (Participant 4, 23 years old)

“The app is open to everyone and it’s good because of the privacy; once you get to test yourself and know your status, it will only be between you and your family; another thing is that the app can encourage me and remind me I’m not alone; so, the app can give you strength.” (Participant 24, Female 31 years old)

Participants demonstrated high coherence of the tool (CF = 50, 83% participants), showing clear understanding of how the BeSure™ application could achieve its aim of enhancing HIV service delivery. Participants reported specific features of the tool and outlined its function. High coherence was observed with similar proportions across younger and older participants.

“I would follow up on my (HIV) status, because sometimes you can miss your appointments.” (Participant 25, Female 20 years old)

“There are counselors, so if I have issues, (BeSure) allows you to call someone and talk to them.” (Participant 17, Female 28 years old)

“Even be able to educate others that it’s not mandatory that you to the hospital to get tested but you can do it for yourself and guide them (with BeSure).” (Participant 6, Female 36 years old)

The majority of the participants considered the application to have low technical burden for use (CF = 42, 58% participants). After learning about BeSure™, participants mentioned it would be easy to learn and operate in daily life. One participant noted that its accessibility and video resources could enable illiterate clients to use the tool.

“As long as I am someone who is used to operating a smartphone, I don’t think it can be hard to me.” (Participant 17, Female 28 years old)

“There are video resources that they can watch, step-by-step.” (Participant 14, 25 years old)

Few participants (CF = 3, 8% participants) commented specifically on the low opportunity cost of using the tool. That being said, two female participants highlighted potential savings generated by the tool in access to HIV testing services.

“It is cheaper, instead of travelling to the hospital, I could just buy data and know more about (the services).” (Participant 6, Female 36 years old)

“It is easier than telling (my partner) to miss work to go get tested.” (Participant 2, Female 23 years old)

The majority of participants reported high perceived effectiveness of the tool (CF = 17, 58% participants), highlighting how the tool could achieve health or social outcomes. Many participants emphasized how the digital tool could enhance HIV testing. High perceived effectiveness of the tool was reported by most young participants (75%) but by fewer than half of the older participants (42%). Notably, older participants mentioned the effectiveness of the tool to empower community access to care beyond just the individual.

“It conserves people’s privacy and so makes it…easier to get tested.” (Participant 17, 28 years old)

“Refer someone else to the nearest place where they can get the services they need.” (Participant 16, 42 years old)

#### Comparison to digital health landscape

In general, participants reported limited awareness of digital health technologies. When probed, participants shared diverse areas in which they had encountered DHTs in Kenya, including tools for pharmaceuticals, emergency services, electronic medical records, and wearable technologies. Two young adult participants mentioned MyDawa™ (24), a pharmaceutical delivery service. Another young male highlighted a Red Cross application, which was primarily used for emergency medical services. Another participant highlighted the KenyaEMR platform, used for health data storage. Two female participants mentioned wearable health technologies, though they noted these technologies were not widespread in Kenya. One participant specifically highlighted that exposure to DHT is low in Kenya, and that the country is not fully leveraging existing technologies.

“With MyDawa we ordered some medicine and was delivered (by MyDawa).” (Participant 4, Male 23 years old)

“(Red Cross app) is mainly for booking; maybe you have an event and you need a team of first aid.” (Participant 14, Male 25 years old)

“‘Kenya EMR’ is an online platform where health data is entered for clients.” (Participant 16, Female 42 years old)

“I see people can be able to monitor their health especially those with disorders such as pressure, when they wear those smart buds.” (Participant 7, 29 years old)

“So, we have the gadgets and we don’t know how powerful they are.” (Participant 23, Female 23 years old)

## Discussion

The primary aim of this study was to explore perceptions of the acceptability of digital health technologies for HIV testing service enhancement, using BeSure™ as an example, and to identify determinants of theoretical use. Key findings included widespread acceptability of the tool across multiple constructs. However, there was also widespread lack of awareness of the tool and other DHTs, perceived to be driven by inadequate and inappropriate marketing, structural barriers, and apathy toward health-related matters. There were several barriers to theoretical use of the tool, including cost of and access to internet services and HIV-associated stigma. Finally, it was noted that participants had limited knowledge of and exposure to digital health tools in their everyday lives.

Participants found DHT in general, and BeSure™ specifically, to be highly acceptable for HIV testing enhancement across age groups. Applying Sekhon and Cartwright's TFA constructs (22), participants reported a positive affect toward the tool, high coherence of its utility, and perceived it as effective, with low burden for use and alignment with their value system. These findings reinforce prior studies showing widespread acceptability of patient-facing DHTs in Kenya among vulnerable groups (25–28), though literature assessing DHT use in emergency care populations remains limited. EDs may serve as a venue to reach “the last mile” with HIV programming in LMICs, given their contact with patients of higher-risk profiles (15). Therefore, the widespread acceptability of a DHT for HTS within the emergency care population supports this potential.

Furthermore, the acceptability of DHTs in this subpopulation aligns well with the current Kenyan national health policy direction. DHT use in this subpopulation may support the Ministry of Health's 2023–2030 HIV prevention strategies, including implementing a precision approach based on vulnerability and risk, as well as integration of HIV prevention into essential services such as emergency care (29). Our findings further support the goal of Kenya's recently established Digital Health Agency, tasked with integrating e-health solutions into delivery of health services (18). Our findings further highlight key areas to address to better harness the potential of DHTs in this setting, highlighting the need for continued implementation studies assessing adoption, integration into routine health services, and alignment with national systems.

Participants highlighted several barriers to address and facilitators to leverage for the theoretical use of BeSure™ as an example DHT for HTS. A key barrier was the lack of widespread and strategic marketing, which limited awareness of the tool. Participants highlighted that promoting within health facilities could be effective, but should strategically consider timing, location, and personnel involved in the promotion. While emergency care venues hold theoretical value for HIV prevention interventions—given that emergency patients tend to be at higher risk of HIV (15)—participants highlighted the challenges in promoting DHT in this important setting, given the acuity of illness or injury.

Yew et al. recently published a systematic review identifying barriers and facilitators to DHT implementation in LMIC health systems and similarly reported the importance of incorporating DHTs during moments where patients and staff can meaningfully engage in the technology (30). Lessons from global demand creation strategies may be applied to digital tools for HTS in emergency care, mobilization, couple- and motivation-oriented counseling, peer-led interventions, conditional fixed-value incentives, and SMS reminders (31). Participants highlighted apathy toward health matters as a barrier to building awareness and use, suggesting that targeted user design or structured incentives could help overcome this challenge, consistent with findings from related studies (32). Addressing barriers and leveraging facilitators identified in this study while learning from previous work could improve future implementation of DHTs for HTS in emergency care populations.

While digital literacy and access to smartphones were not emphasized as barriers in this sample of urban young adults, cost of data and internet were repeatedly cited. These findings underscore the need for sustainable funding mechanisms for digital health interventions, as outlined in Kenya’s recently passed Digital Health Act (18), as well as structural interventions to increase access to the internet across the country, such as Starlink's expansion in Kenya in 2024 (33). Stigma was highlighted as a key modifier to use of the BeSure™ technology. Some participants valued the tool for its protection of privacy and anonymity, while others feared that use of the tool itself could be stigmatizing. This duality emphasizes the importance of continuing to acknowledge, study, and address persisting stigma in HIV testing interventions in Kenya (34). Moreover, perhaps a DHT with a broader focus beyond just HIV may help maintain desired privacy, as demonstrated in an assessment of a digital health intervention among Kenyan female sex workers (35).

Several thematic differences were observed between older and younger participants that may assist in better targeting tools like BeSure™. In the sample, more young adult participants made positive comments about the tool and the vast majority perceived the tool be very acceptable. Young adult participants were more likely to mention the use of social media, including TikTok, Instagram, and Facebook, as facilitators, reflecting high smartphone and internet penetration (∼96% population) in Kenya (33). They were also more likely to report perceiving HIV risk as a facilitator, which correlates with epidemiologic trends of the increasing incidence of new infections among Kenyan youth (1). Targeting young adults with a digital HTS tool in Kenyan emergency care may therefore be best implemented by leveraging social media platforms (36, 37). No clear thematic differences were observed across genders in this study, suggesting the need for further exploration into gender-specific targeting or perception of these technologies within emergency care populations.

This study had several limitations. First, BeSure™ was not accessible during the data collection period, limiting our ability to get real-time user experience data and thus ecological validity. However, crafting of the interview guide took this into account, and our questions assessed awareness and use of BeSure™ as an example DHT, while also focusing on the broader digital health landscape, thereby improving the generalizability of our findings. In addition, conducting such acceptability research while a tool is offline is not uncommon in digital health, where findings can inform successful tool relaunch. Selection bias was present given that the sample comprised urban youth with significant digital literacy and familiarity with smartphones. This degree of digital literacy may not be present across all Kenyan populations, though high internet penetration suggests that generalizability is not limited. Moreover, the target user audience of the tool includes those with smartphones. While differences in perceptions between older and younger adult participants emerged, more than 70% of participants were younger than 30 years old, limiting insights into of this tool among those over than 45 years. However, most emergency care patients in LMICs are youth (38), suggesting that our findings are likely generalizable for DHTs implemented for these populations. Finally, per the design of this study, participants were enrolled from a broader study wherein they had been exposed to an HIV testing intervention in an ED (21), potentially limiting generalizability to groups without such exposure. Nonetheless, the vast majority of participants interviewed did not recall undergoing the HIV testing intervention.

Ultimately, there was widespread acceptance of the BeSure™ tool specifically, and of DHTs for HTS enhancement more broadly, within emergency care populations. The landscape for DHTs for HIV programming is expanding in Kenya, making it critical to understand acceptability, identify determinants of use, and assess impact ahead of scaled implementation. As the HIV epidemic continues to become concentrated in subpopulations in Kenya and across Africa (1), viable digital health technologies like BeSure™ may be useful in targeting resources for precision prevention of HIV.

## Data Availability

The raw data supporting the conclusions of this article will be made available by the authors, without undue reservation.

## References

[1] Joint United Nations Programme on HIV/AIDS (UNAIDS). The Urgency of Now: AIDS at a Crossroads—Global AIDS Update 2024. Geneva: UNAIDS (2024). Available online at: https://www.unaids.org/en/resources/documents/2024/global-aids-update-2024 (Accessed December 5, 2025).

[2] Black MC Basile KC Breiding MJ Smith SG Walters ML Merrick MT The National Intimate Partner and Sexual Violence Survey (NISVS): 2010 Summary Report. Atlanta, GA: National Center for Injury Prevention and Control, Centers for Disease Control and Prevention (2011).

[3] Reisner SL Poteat T Keatley J Cabral M Mothopeng T Dunham E Global health burden and needs of transgender populations: a review. Lancet. (2016) 388(10042):412–36. 10.1016/S0140-6736(16)00684-X 27323919 PMC7035595

[4] Valencia J Alvaro-Meca A Troya J Gutiérrez J Ramón C Rodríguez A Gender-based vulnerability in women who inject drugs in a harm reduction setting. PLoS One. (2020) 15(3):e0230886. 10.1371/journal.pone.0230886 32226042 PMC7105126

[5] Forson PK Oduro G Bonney J Cobbold S Sarfo-Frimpong J Boyd C Emergency department admissions Kumasi, Ghana: prevalence of alcohol and substance use, and associated trauma. J Addict Dis. (2020) 38(4):520–8. 10.1080/10550887.2020.1791378 32664825

[6] Aboagye RG Mireku DO Nsiah JJ Ahinkorah BO Frimpong JB Hagan JE Prevalence and psychosocial factors associated with serious injuries among in-school adolescents in eight sub-Saharan African countries. BMC Public Health. (2022) 22(1):853. 10.1186/s12889-022-13198-6 35484506 PMC9047327

[7] Aluisio AR Rege S Stewart BT Kinuthia J Levine AC Mello MJ Prevalence of HIV-seropositivity and associated impact on mortality among injured patients from low-and middle-income countries: a systematic review and meta-analysis. Curr HIV Res. (2017) 15(5):307–17. 10.2174/1570162X15666170920112743 28933280

[8] Poxon ML A McDermott M Kariri A Kaul R Kimani J. Emergency departments as under-utilized venues to provide HIV prevention services to female sex workers in Nairobi, Kenya. ResearchSquare [Preprint]. (2022). 10.21203/rs.3.rs-2274148/v1 PMC10399019 37537558

[9] Aluisio AR Sugut J Kinuthia J Bosire R Ochola E Ngila B Assessment of standard HIV testing services delivery to injured persons seeking emergency care in Nairobi, Kenya: a prospective observational study. PLoS Global Public Health. (2022) 2(10):e0000526. 10.1371/journal.pgph.0000526 36962519 PMC10021732

[10] Hansoti B Stead D Eisenberg A Mvandaba N Mwinnyaa G Patel EU A window into the HIV epidemic from a South African emergency department. AIDS Res Hum Retroviruses. (2019) 35(2):139–44. 10.1089/aid.2018.0127 30215268 PMC6360397

[11] Peltzer K Matseke G. Determinants of HIV testing among young people aged 18–24 years in South Africa. Afr Health Sci. (2013) 13(4):1012–20. 10.4314/ahs.v13i4.22 24940326 PMC4056506

[12] Delany-Moretlwe S Cowan FM Busza J Bolton-Moore C Kelley K Fairlie L. Providing comprehensive health services for young key populations: needs, barriers and gaps. J Int AIDS Soc. (2015) 18(2 Suppl 1):19833. 10.7448/ias.18.2.19833 25724511 PMC4344539

[13] McGuire M de Waal A Karellis A Janssen R Engel N Sampath R HIV self-testing with digital supports as the new paradigm: a systematic review of global evidence (2010–2021). eClinMed. (2021) 39:101059. 10.1016/j.eclinm.2021.101059 PMC8367787 34430835

[14] Joint United Nations Programme on HIV/AIDS. DANGER: UNAIDS global AIDS update 2022. Geneva (2022). 12349391

[15] Smith-Sreen J Bosire R Farquhar C Katz DA Kimani J Masyuko S Leveraging emergency care to reach key populations for ‘the last mile’ in HIV programming: a waiting opportunity. Aids. (2023) 37(15):2421–4. doi: 10.1097/qad.0000000000003709. 37965739 10.1097/QAD.0000000000003709 PMC10655840

[16] National AIDS/STI Control Programme (NASCOP), Ministry of Health, Kenya. National Implementation Guidelines for HIV and STI Programming Among Young Key Populations. Nairobi: Government of Kenya (2018).

[17] Boothe MAS Sema Baltazar C Sathane I Raymond HF Fazito E Temmerman M Young key populations left behind: the necessity for a targeted response in Mozambique. PLoS One. (2021) 16(12):e0261943. 10.1371/journal.pone.0261943 34972172 PMC8719759

[18] National Council for Law (NCfL). The Digital Health Act. Nairobi: NCfL (2023).

[19] National AIDS & STI Control Programme (NASCOP). BeSure Kenya (2017). Available online at: https://www.besure.co.ke/ (Accessed December 5, 2023).

[20] World Health Organization. Monitoring and Evaluating Digital Health Interventions: A Practical Guide to Conducting Research and Assessment. Geneva: World Health Organization (2016). Available online at: https://www.who.int/publications/i/item/9789241511766 (Accessed December 5, 2025).

[21] Aluisio AR Smith-Sreen J Offorjebe A Maina W Pirirei S Kinuthia J Assessment of the HIV enhanced access testing in the emergency department (HEATED) program in Nairobi, Kenya: a quasi-experimental prospective study. HIV Res Clin Pract. (2024) 25(1):2403958. 10.1080/25787489.2024.2403958 39290079 PMC11443818

[22] Sekhon M Cartwright M Francis JJ. Acceptability of healthcare interventions: an overview of reviews and development of a theoretical framework. BMC Health Serv Res. (2017) 17(1):88. 10.1186/s12913-017-2031-8 28126032 PMC5267473

[23] Sekhon M Cartwright M Francis JJ. Acceptability of health care interventions: a theoretical framework and proposed research agenda. Br J Health Psychol. (2018) 23(3):519–31. 10.1111/bjhp.12295 29453791

[24] MyDawa – we deliver care (2025). Available online at: https://mydawa.com/ (Accessed December 5, 2023).

[25] Mills R Krong R Kithinji F Baraitser P. Digital training for self-injectable contraceptives: a feasibility and acceptability pilot study. BMJ Sex Reprod Health. (2024) 51(2):144–6. 10.1136/bmjsrh-2023-202197 39160059

[26] Soehnchen C Weirauch V Schmook R Henningsen M Meister S. An acceptance analysis of a sexual health education digital tool in resource-poor regions of Kenya: an UTAUT based survey study. BMC Womens Health. (2023) 23(1):676. 10.1186/s12905-023-02839-6 38114976 PMC10729446

[27] Kiburi SK Paruk S Kwobah EK Chiliza B. Exploring user experiences of a text message-delivered intervention among individuals on opioid use disorder treatment in Kenya: a qualitative study. PLOS Digit Health. (2023) 2(11):e0000375. 10.1371/journal.pdig.0000375 37930956 PMC10627438

[28] Kiburi SK Paruk S Chiliza B. Mobile phone ownership, digital technology use and acceptability of digital interventions among individuals on opioid use disorder treatment in Kenya. Front Digit Health. (2022) 4:975168. 10.3389/fdgth.2022.975168 36093384 PMC9452845

[29] National Syndemic Diseases Control Council (NSDCC), Government of Kenya. National Multisectoral HIV Prevention Acceleration Plan 2023–2030. Nairobi: NSDCC (2023).

[30] Yew SQ Trivedi D Adanan NIH Chew BH. Facilitators and barriers to the implementation of digital health technologies in hospital settings in lower- and middle-income countries since the onset of the COVID-19 pandemic: scoping review. J Med Internet Res. (2025) 27:e63482. 10.2196/63482 40053793 PMC11926458

[31] Wagner AD Njuguna IN Neary J Lawley KA Louden DKN Tiwari R Demand creation for HIV testing services: a systematic review and meta-analysis. PLoS Med. (2023) 20(3):e1004169. 10.1371/journal.pmed.1004169 36943831 PMC10030044

[32] Peng R Chang J Du Y Zhang C Li X Guo Y Older adults’ perceptions and experiences of engaging in web- and mobile-based physical activity interventions: a systematic review and qualitative meta-synthesis. Geriatr Nurs (Minneap). (2024) 59:630–8. 10.1016/j.gerinurse.2024.08.025 39197354

[33] Communications Authority of Kenya. Third Quarter Sector Statistics Report, Financial Year 2023/2024. Nairobi: Communications Authority of Kenya (2024).

[34] Agot K Cain M Medley A Kimani J Gichangi P Kiio C Formative assessment to identify perceived benefits and barriers of HIV oral self-testing among female sex workers, service providers, outreach workers, and peer educators to inform scale-up in Kenya. AIDS Care. (2022) 34(6):717–24. 10.1080/09540121.2021.1894318 33657929 PMC10962321

[35] Ampt FH L'Engle K Lim MSC Plourde KF Mangone E Mukanya CM A mobile phone–based sexual and reproductive health intervention for female sex workers in Kenya: development and qualitative study. JMIR Mhealth Uhealth. (2020) 8(5):e15096. 10.2196/15096 32469326 PMC7293053

[36] Mason S Ezechi OC Obiezu-Umeh C Nwaozuru U BeLue R Airhihenbuwa C Understanding factors that promote uptake of HIV self-testing among young people in Nigeria: framing youth narratives using the PEN-3 cultural model. PLoS One. (2022) 17(6):e0268945. 10.1371/journal.pone.0268945 35657809 PMC9165856

[37] Cao B Gupta S Wang J Hightow-Weidman LB Muessig KE Tang W Social media interventions to promote HIV testing, linkage, adherence, and retention: systematic review and meta-analysis. J Med Internet Res. (2017) 19(11):e394. 10.2196/jmir.7997 29175811 PMC5722976

[38] Obermeyer Z Abujaber S Makar M Stoll S Kayden SR Wallis LA Emergency care in 59 low- and middle-income countries: a systematic review. Bull World Health Organ. (2015) 93(8):577–86. 10.2471/blt.14.148338 26478615 PMC4581659

