# Supplementary material for: Acceptability and use determinants of digital health technologies for HIV services: a qualitative study of emergency care patients in Nairobi, Kenya
Source: Front Digit Health. 2026 Jan 23;7:1697814. doi: 10.3389/fdgth.2025.1697814 (PMC12876169; doi:10.3389/fdgth.2025.1697814)
Supplement: Supplementary file 1 [file Supplementaryfile1.docx]

**Supplementary Material 1**: Bilingual pamphlet provided to participants describing the BeSure^TM^ technology

**
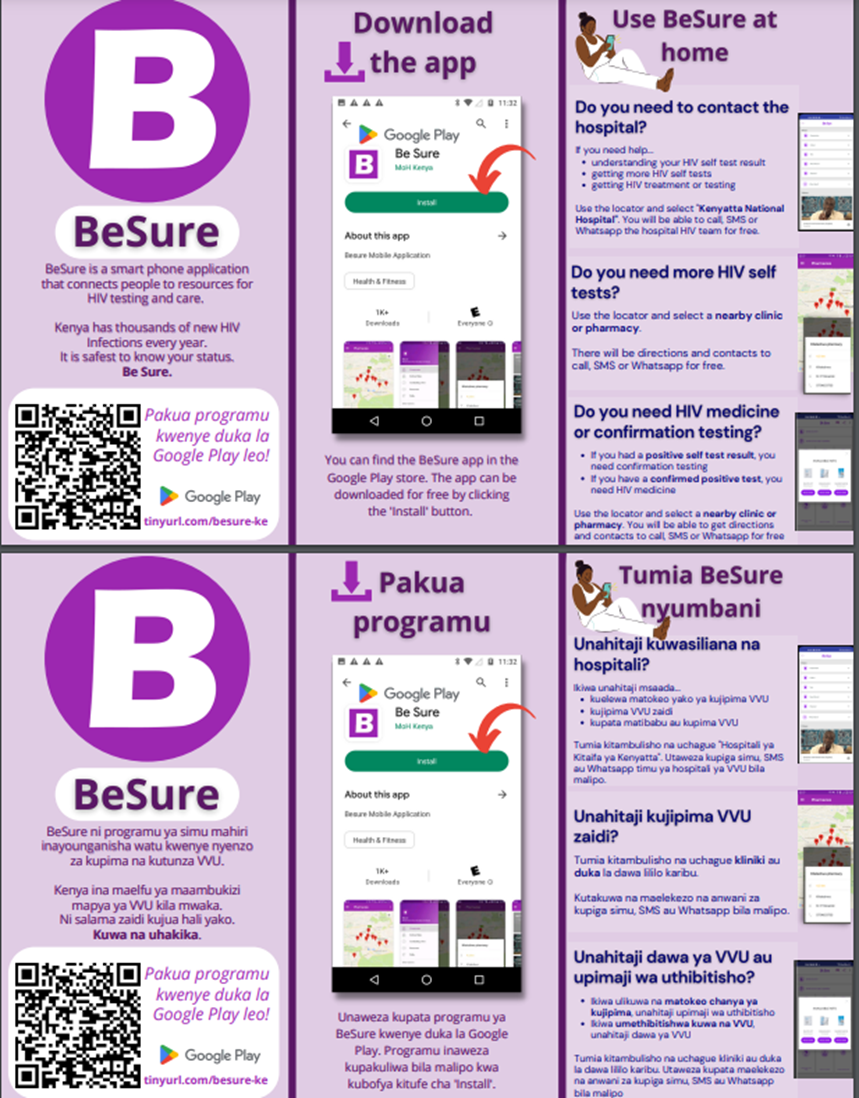
**
