# Supplementary material for: Acceptability and use determinants of digital health technologies for HIV services: a qualitative study of emergency care patients in Nairobi, Kenya
Source: Front Digit Health. 2026 Jan 23;7:1697814. doi: 10.3389/fdgth.2025.1697814 (PMC12876169; doi:10.3389/fdgth.2025.1697814)
Supplement: Supplementary file 2 [file Supplementaryfile2.docx]

**Supplementary Material 2**: In-Depth Interview Guide

HIV in Injury: Evaluation of HIV Testing and Care in African Emergency Care for the Injured (A Pilot Implementation And Evaluation Study Of the HIV Enhanced Access Testing Program in An Emergency Care Setting in Kenya

**PRE-INTERVIEW INTRODUCTION**

**Introductions**

Good [morning/afternoon]. My name is _____________. Thank you so much for agreeing to come in to have an interview. I also want to recognize that you were a participant in our study during your time in the KNH Accidents & Emergencies, and that you agreed to be followed up by phone. We sincerely thank you for participating thus far and hope your recovery from your injury has been okay.

**Informed Consent**

Please see here the informed consent document. Before we begin with the interview, we will have to complete the informed consent. We obtain your signature so that you fully understand and agree to the interview.

**Description of study**

Again, I know you participated in our study here at the KNH A&E, but I wanted to ensure you understand what the study is about. We are trying to understand the experience of injury patients receiving HIV testing services in the A&E – whether they were offered testing, whether they received testing, whether they took home a self-test. We are very grateful to you for sharing your information– it is very valuable to understand HIV testing in the A&E. Today, we are interested in your thoughts on and experiences with digital health tools as a way to help people increasing access to HIV testing services either in facilities or through using an HIV self-tests. In the A&E, you might have heard about one digital health tool called BeSure – a platform made in Kenya that is used to provide information and resources on HIV testing. We want to understand your thoughts and beliefs on BeSure and digital health tools in general that can help people learn about and complete HIV testing.

**Description of digital health tools**

Do you know what I mean when I say a digital health tool? A digital health tool is something like a website or a smartphone application that is designed to focus on healthcare topics. These digital health tools can be on different platforms, which means on the internet as a website or on a smartphone as an application, or “app”. M-PESA is a platform that does this in that it can work on a website or on your phone to help you store and send money, and like M-PESA a digital health tool can be on a phone or a website and will help you with something about your health.

**Description of BeSure (*provide BeSure pamphlet*)**

As I mentioned ,ne example of a digital health tool is the BeSure platform which you may have heard about in the A&E. The Ministry of Health in Kenya created the BeSure platform, which can be accessed on your phone, by download from the Google Play Store, or on your computer with a website. BeSure tries to help people to access HIV testing, talk to someone at a hospital about HIV testing (through Whatsapp numbers), use HIV self-tests at home with informational videos, and find clinics near them for HIV services – like getting medicines, getting more tests or just learning more. BeSure is not currently available because it is being updated by the Ministry of Health, but we wanted to understand your thoughts about digital health tools in general - the BeSure application is just one example.

Do you have any questions for me?

If not, we can now begin the interview. With your consent, I will begin recording.

**In-depth interview Agenda**

| **Part 1: INTRODUCTIONS / DEMOGRPAHICS (USERS AND NON-USERS)** |
| --- |
| Intent: *To open the interview and understand the identity of the participant. This will serve to establish comfort and rapport with the interviewer. It will be important to use the time here to help the patient understand what a qualitative interview is like (i.e., not an interrogatory question-answer format, but a conversation that flows naturally. Interviewer should provide empathy when participant shares challenges related to their injury and/or identities.*  Question 1: Tell me what happened when you came to the A&E department after your injury?   - Probes:   - What types of providers took care of you during the A&E visit?     - What did each of them do for you?   - What well during the A&E visit?   - What did not go well? - Prompts:   - *“tell me more specifically what happened: take me through it step-by-step”*   - *“what did you think about the part of you’re A&E experience”*   - *“how did you feel about that specific part of your A&E experience”* |
| **Part 2: SCREEN (USERS AND NON-USERS)** |
| Screen: *To assess whether the participant has used the BeSure digital health tool and mobile health application. All participants should have at least heard of the platform after exposure to HEAT programming based on previous questions, but participants may not remember what the platform is.*  Question 2: Have you used the BeSure platform before?   - Probes:   - *Have you heard of the BeSure platform before?*   - *Have you used any digital health platform before?*   - When I ask whether you have used the platform, I am asking whether you have entered the platform on your phone or on a computer, to get support on using your self-tests, getting connected to clinics for testing or treatment, or for any other reason. - Prompts*:*    - *“The platform was made to to increase access to HIV testing and treatment; you can access the platform on the Internet using your phone or on a computer”*     - *Mention that the Besure platform is one example of a digital health tool that aims to increase access and information about HIV testing services, specifically HIV self tests. Mention that it provides information on tests, and provides Whatsapp numbers of clinicians and locations of clinics where testing is provided.*   - *Show participant the pamphlet of the platform to prompt* |
| **Part 4A: AWARENESS (NON-USERS)** |
| Intent: *To understand the awareness of the application by the participant, and identify and perceived barriers for those who may not have been aware of the application. Again, ask this hypothetically or based on any used digital health platforms if they have never heard of or used BeSure.*  Question 3: How did you first hear about the BeSure platform?   - **Probes**:   - Had you heard about the BeSure platform before your visit to the A&E?     - If yes,       - where did you hear about it?       - did you use it before? Why or why not?       - What did you think the purpose of the platform was when you first heard about it?     - If no,       - why do you think you did not hear about the platform before?       - what do you think would have been helpful to have made you aware of the platform before your visit to the A&E?     - BeSure is one example of a digital health platform for HIV information and testing support. Have you heard about any other digital health platforms for HIV testing?       - If yes, please share more about the platforms and your experience |
| **Part 4B: ACCESS (NON-USERS)** |
| Intent: *To understand and identify barriers to use for this population and perceived facilitators for those who did use the platform. Again, ask this hypothetically or based on any used digital health platforms if they have never heard of or used BeSure.*  Question 4: Tell me more about why you did not use the platform.   - Probes:   - Tell me more about what was shared with you during your visit to the A&E visit about the BeSure platform.     - Is there anything the staff mentioned that made you less interested in using the platform?   - Tell me about your access to a mobile phone, data and/or a computer. Did this prevent you from using the platform?   - What other factors may have been a barrier to using the application?   - Why do you think some people did use the application?   - Do you have any ideas or suggestions about how to promote awareness or use of the platform?     - What would have been helpful for you to have used the platform?     - BeSure platform that is a website and an application you can download from the Google Play Store.       - Do you think a website or an “app” would be the best way for you to get information and locations for HIV testing?     - BeSure platform provides Whatsapp numbers to contact clinicians that can help with HIV testing.       - Do you think having Whatsapp numbers – and being able to talk to people on the phone, is the best way for you to get information about HIV testing?     - Other digital health platforms exist where there is a “chatbot” for contact, where you can get send a message to a Whatsapp number, and it instantly responds with information. This is not a “real person”, but is artificial intelligence that can provide you some information on HIV testing instantly. Would this be a better way to get information on HIV testing? If you could choose anything or design your own digital health tool for HIV testing, what type of platform would you want? What would it look like? - Prompts:   - *“tell me more about that”*   - *“why do you think providers feel that way”*   - *“what are the concerns you have about that”* |
| **Part 7: CONCLUSION** |
| Intent*: This is a time to provide an additional chance for the participant to give any thoughts for the topics of interest and for the facilitator to follow up on any discussion on the topics brought forth during the interview that could be supported by more information.*  Question 8: Are there any other thoughts you have about the BeSure platform that are important to consider that we have not discussed today?   - Prompts:   - *“tell me more about that”*   - *“why would that option be preferred”* |
